# FOXO1 downregulation contributes to the oncogenic program of primary mediastinal B-cell lymphoma

**DOI:** 10.18632/oncotarget.2107

**Published:** 2014-06-15

**Authors:** Linka Xie, Olga Ritz, Frank Leithäuser, Hanfeng Guan, Johanna Färbinger, Clarissa D. Weitzer, Franziska Gehringer, Silke Brüderlein, Karlheinz Holzmann, Marion J. Vogel, Peter Möller, Thomas Wirth, Alexey Ushmorov

**Affiliations:** ^1^ Cancer Center of Union Hospital, Tongji Medical College, HuaZhong University of Science and Technology, Wuhan, China; ^2^ Institute of Physiological Chemistry, University of Ulm, Germany; ^3^ Institute of Pathology, University of Ulm, Germany; ^4^ Department of Orthopaedic Surgery, Tongji Hospital, Tongji Medical College, Hua Zhong University of Science and Technology, Wuhan, China; ^5^ Genomics Core Facility University of Ulm, Germany

**Keywords:** primary mediastinal B cell lymphoma, FOXO1, JAK2, BCL2L1/BCLxL, MYC

## Abstract

Recently we have shown that the transcription factor FOXO1, highly expressed in B cells, is downregulated in classical Hodgkin lymphoma (cHL). As primary mediastinal B cell lymphoma (PMBL) has similarities with the cHL transcription program we investigated FOXO1 expression in this entity. By using immunohistochemistry we found that FOXO1 was absent or expressed at low levels in 19 of 20 primary PMBL cases. PMBL cell lines reproduce the low FOXO1 expression observed in primary cases. By analyzing gene expression profiling data we found that *FOXO1* expression inversely correlated with *JAK2* in PMBL cases. Targeting JAK2 activity by the small molecular weight inhibitor TG101348 resulted in upregulation of FOXO1 mRNA and protein expression in MedB-1 and U2940 cell lines, and the MYC inhibitor 10058-F4 increased *FOXO1* mRNA in MedB-1 cells. Moreover, in MedB-1 cells FOXO1 expression was strongly upregulated by the inhibitor of DNA methylation 5-aza-2-deoxycytidine and by the histone deacetylase inhibitor trichostatin A. Since *FOXO1* promoter was unmethylated, this effect is most likely indirect. FOXO1 activation in the FOXO1-negative MedB-1 cell line led to growth arrest and apoptosis, which was accompanied by repression of MYC and BCL2L1/BCLx_L_. Thus, FOXO1 repression might contribute to the oncogenic program and phenotype of PMBL.

## INTRODUCTION

PMBL is a distinct subtype of diffuse large B cell lymphoma (DLBCL) sharing several morphological and molecular similarities with classical Hodgkin lymphoma (cHL). It comprises about 2% of NHL and preferentially affects young females [1]. Although PMBL belongs to the most curable lymphoma subtypes [2], delayed late complications of chemo- and radiotherapy remain a main challenge. With respect to complications of chemotherapy such as cardiomyopathy after high anthracycline doses and mediastinal irradiation, further investigation of the PMBL oncogenic program to find specific molecular targets is warranted.

The oncogenic program of PMBL is similar to that of cHL. Both entities rely on constitutive activation of JAK-STAT and NF-κB signaling [3]. The genomic amplifications on 9p and 2p16 involving *JAK2* and *REL* genes, respectively, are recurrent features of PMBL and cHL [4, 5]. Furthermore, suppressor of cytokine signaling 1 (SOCS1), a negative regulator of JAK/STAT signaling, is recurrently mutated in both entities leading to increased phosphorylation of the JAK2 downstream targets STAT5 and STAT6 [6]. STAT transcription factors, in turn, induce transcription of genes responsible for proliferation and survival including *MYC* and *BCLx_L_/BCL2L1*[7]. In addition, JAK2 and histone demethylase KDM4/JMJD2C can directly activate *MYC* transcription in PMBL and cHL cell lines [8].

Despite these similarities, PMBL principally differ from cHL, e.g. in terms of maintenance of major parts of the B cell differentiation program. The characteristic trait of cHL is almost complete loss of the B cell phenotype, whereas PMBL express most of the B cell-specific transcription factors including POU2AF1/BOB.1/OBF1, POU2F2/OCT2, PU.1, PAX5, BCL6 and B cell surface differentiation markers CD19, CD20, and CD79a [9]. However, PMBL like cHL typically lacks surface immunoglobulins [10].

Recently, we have shown that the forkhead O family transcription factor FOXO1, which is highly expressed in B cells, is downregulated in Hodgkin and Reed-Sternberg (HRS) cells of cHL. Interestingly, all NHL subtypes tested including follicular lymphoma, marginal zone B-cell lymphoma, DLBCL, marginal zone B lymphoma of mucosa-associated lymphoid tissue, B-cell chronic lymphocytic leukemia, mantle cell lymphoma, and Burkitt lymphoma expressed FOXO1 protein at levels comparable with those of normal B cells [11]. FOXO family transcription factors have been shown to act as tumor suppressors regulating expression of proapoptotic and antiproliferative genes [12]. FOXO1 plays a critical role in establishing and maintaining the B cell specific differentiation program, but it is also responsible for cell death due to an inappropriate BCR signaling [13, 14]. The best-studied mechanism of FOXO inactivation is phosphorylation followed by nuclear export and proteolytic degradation. AKT, ERK, and IKK kinases are known to phosphorylate FOXO proteins thereby contributing to cell proliferation and survival [15-18]. Constitutive activation of PI3K/AKT and ERK pathways is typical for many lymphoma subtypes [19, 20]. In addition, FOXO1 mutations were detected in 7% of all NHLs [21] and in 8.6% cases of DLBCL. These mutations did not influence FOXO1 mRNA and protein levels [22]. In cHL high expression of specific miRNAs, chromosomal deletions, and constitutive activity of AKT and ERK signaling pathways contribute to almost complete repression of FOXO1 [11].

Considering that PMBL resembles cHL in various aspects, we asked whether it also expresses low levels of FOXO1 and which role FOXO1 might play in PMBL. By using immunohistochemistry we found that most PMBL cases were either low or negative for FOXO1. We identified FOXO1 as a tumor suppressor in PMBL and revealed mechanisms responsible for its repression.

## RESULTS

### FOXO1 is repressed in PMBL

**Figure 1:**
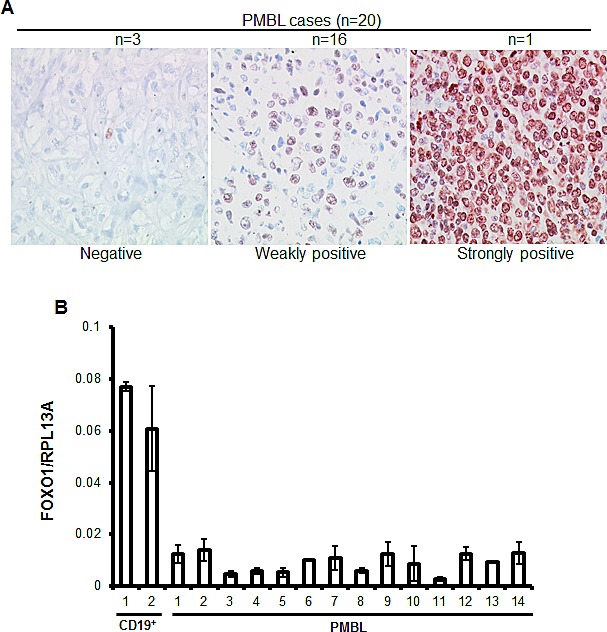
FOXO1 is downregulated in PMBL (A) High-magnification images show differential FOXO1 expression in malignant cells of PMBL (original magnification ×200). Objective: Plan-Neofluar 40×/0.75 NA. (B) FOXO1 mRNA expression is downregulated in PMBL cases. RNA was isolated from CD19^+^ tonsillar cells and from frozen PMBL cases, and *FOXO1* expression was measured by Q-RT-PCR and analyzed by delta Ct method. Here and in all other Q-RT-PCR experiments the house keeping gene *RPL13A* was used as reference and the data are shown as mean±SD of at least 2 independent Q-RT-PCR runs that included 3 technical replicates each.

**Figure 2:**
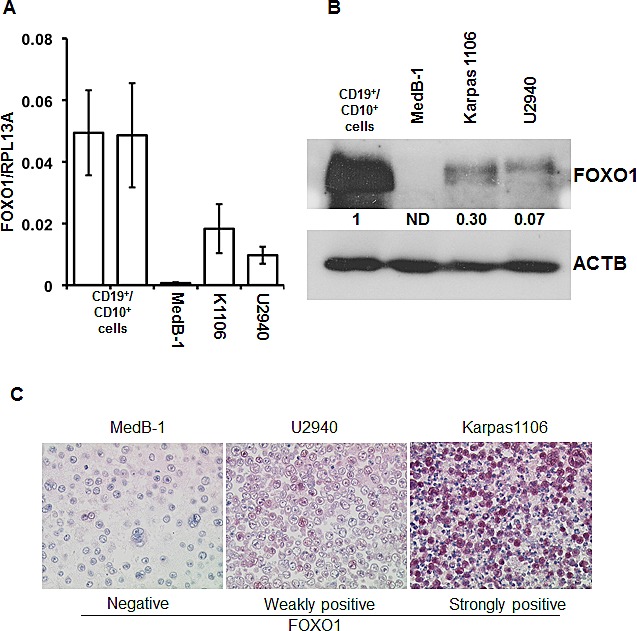
FOXO1 expression differs in PMBL cell lines (A) FOXO1 mRNA expression in PMBL cell lines and in CD19^+^/CD10^+^ tonsillar cells was measured by Q-RT-PCR. The data are shown as mean±SD FOXO1/PRL13A ratio calculated by delta Ct method. The total RNA was isolated from at least three samples of each cell line. (B) Expression of FOXO1 protein in PMBL cell lines and in CD19^+^/CD10^+^ tonsillar cells was analyzed by immunoblot. ACTB was used as loading control. Here and in the following immunoblot images the representative of at least three analyses of protein lysates of cells harvested at different time points of culturing are shown. All immonoblot analyses yielded similar results. (C) FOXO1 expression in paraffin embedded PMBL cell lines. The cells were stained with anti-FOXO1 antibody and microscopy was done as described in legend to Figure 1A. The experiment was repeated two times and yielded identical results.

To clarify the expression status of FOXO1 in PMBL we analyzed 20 clinically and morphologically validated PMBL cases using immunohistochemistry (IHC). In 15% of cases FOXO1 was absent, in 80% of cases only weak staining was observed, and one case (5%) was scored as strongly positive (Figure 1A). Further, we measured expression of *FOXO1* mRNA in an independent PMBL cohort and in two samples of CD19^+^ cells isolated from hyperplastic human tonsils (Figure 1B). The expression of *FOXO1* mRNA in PMBL samples significantly varied but in all cases it was substantially lower than in normal tonsillar B cells. There is a scarcity in cell lines representing PMBL, the only three available cell lines are MedB-1, Karpas1106, and U2949. We therefore analyzed FOXO1 expression in these three PMBL cell lines using Q-RT-PCR, immunoblot, and IHC (Figure 2). The levels of *FOXO1* mRNA in all PMBL cell lines were significantly lower than in CD19^+^/CD10^+^ tonsillar cells representing the germinal (GC) population (Figure 2A). The highest *FOXO1* mRNA levels were detected in Karpas1106, followed by U2940 and MedB-1. Of note, *FOXO1* mRNA expression levels in Karpas1106 and U2940 cells were similar to that in PMBL cases with highest *FOXO1* levels, whereas expression of FOXO1 in MedB-1 cells was somewhat lower than in PMBL cases with lowest expression (Figure 1B and Figure 2A). The FOXO1 protein levels in the PMBL cell lines correlated well with the mRNA data. Karpas1106 expressed the highest and MedB-1 expressed the lowest levels of FOXO1 (Figure 2B). To further corroborate similarities between clinical PMBL cases and cell lines we also used IHC to analyze FOXO1 expression in paraffin embedded pellets of the cell lines (Figure 2C). The cell lines matched well to three groups of primary PMBL tumors which were identified on the basis of FOXO1 expression: FOXO1 negative - MedB-1, weakly positive - U2940, and strongly positive Karpas1106.

### Contribution of genetic and epigenetic factors to FOXO1 repression in PMBL

To clarify the genetic mechanisms of FOXO1 repression we first sequenced both *FOXO1* exons in all three PMBL cell lines (Figure S1). We did not find any missense mutations in the transcribed region and the promoter region of *FOXO1*. Next, we analyzed publically available human genome tiling array data from 31 PMBL cases [28]. We found five samples with copy number aberrations affecting *FOXO1* but none of them was limited to *FOXO1* locus. Specifically, we found one sample with a single allele loss of *FOXO1* and four cases displaying a single allele gain, which was based on trisomy in two cases. Therefore, *FOXO1* copy number aberrations in PMBL are biased to gains rather than to losses and therefore do not contribute to FOXO1 downregulation in this lymphoma subtype (Table S1).

**Figure 3:**
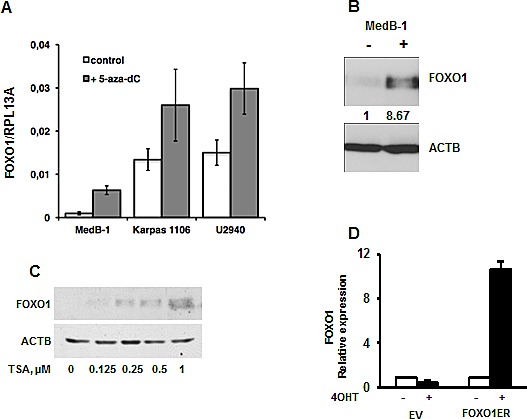
Epigenetic factors contribute to FOXO1 silencing (A, B) PMBL cells were seeded at a density of 2×10^6^ cells in 10 ml of complete culture medium and incubated for 24h with 5-aza-dC at a concentration of 1 μM. The cells were then washed and cultured in complete medium without 5-aza-dC. After 72h, cells were harvested and used for analysis of *FOXO1* mRNA and protein expression by Q-RT-PCR (A) and by immunoblot (B). One of three independent experiments that yielded similar results is shown. The Q-RT-PCR data were analyzed by delta Ct method. (C) MedB-1 cells were treated with graded concentrations of TSA for 24h. FOXO1 protein expression was analyzed by immunoblot. (D) Ectopic FOXO1 activation induces transcription of endogenous FOXO1 in MedB-1 cells. MedB-1 stably expressing inducible variant of FOXO1 (FOXO1ER) or empty vector (EV) were treated with 4-OHT at concentration of 200 nM. 24h later expression of endogenous FOXO1 was measured with help of Q-RT-PCR using primers targeting 3′-UTR. One of three independent experiments that yielded similar results is shown. The data were calculated by comparative Ct method using RPL13A as reference gene.

Epigenetic factors are often involved in repression of tumor suppressor genes. To test the contribution of DNA methylation to the FOXO1 repression we treated PMBL cell lines with the DNA methyltransferase inhibitor 5-aza-dC. We observed an increase of the *FOXO1* mRNA levels in all cell lines with highest upregulation in MedB-1 cells (6.6 fold) whereas in Karpas1106 and U2940 FOXO1 levels were only slightly increased (1.9 and 2 fold, respectively) (Figure 3A). Interestingly, 5-aza-dC increased expression of FOXO1 on protein levels only in MedB-1 (Figure 3B), but not in Karpas1106 and U2940 cell lines (data not shown). Given that histone deacetylation can also be responsible for gene silencing, we treated MedB-1 cells with the histone deacetylase inhibitor TSA. In fact, TSA treatment resulted in a dose dependent increase of FOXO1 protein levels (Figure 3C). Finally, to find out whether the positive effect of 5-aza-dC on FOXO1 expression can be explained by hypermethylation of the *FOXO1* promoter, we analyzed the methylation status of 26 CpG-dinucleotides located in the vicinity of the transcription start site (Figure S2). Surprisingly, the MedB-1 promoter was unmethylated like in normal B cell subtypes (data not shown), suggesting that 5-aza-dC and TSA affect FOXO1 expression indirectly. The absence of promoter methylation indicated “open” chromatin structure of the FOXO1 promoter in MedB-1 cells. To clarify whether this assumption is true, we overexpressed a constitutively active mutant of *FOXO1* fused in frame with the estrogen receptor ligand-binding domain (FOXO1ER) in MedB-1 cells. We measured the expression level of endogenous *FOXO1* mRNA due to the fact that *FOXO1* promoter harbors FOXO binding motifs and is regulated in a positive feed-back loop manner in normal cells [29]. Stimulation with 4-OHT for 24 h increased the levels of *FOXO1* mRNA 8.3 fold in comparison with non-treated control. Thus, the *FOXO1* promoter has functionally “open” chromatin structure and can be activated by unknown epigenetically silenced transcription factors. Taken together, our results suggest that down-regulation of FOXO1 is not due to genetic aberrations in PMBL. Additionally, epigenetic events may play an indirect role in FOXO1 repression.

### FOXO1 expression in PMBL is regulated by a complex network including JAK2 and MYC

**Figure 4:**
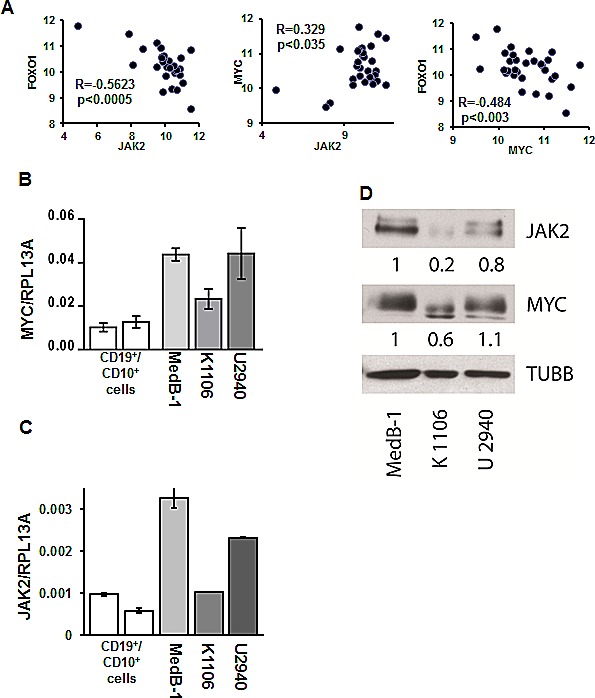

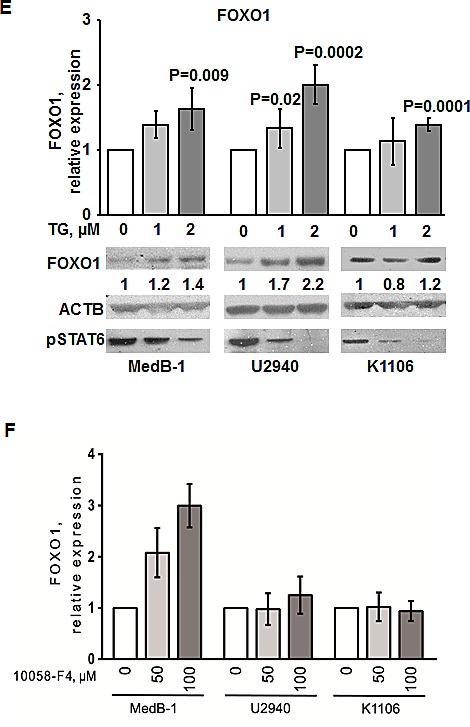
JAK2 and MYC contribute to FOXO1 repression in PMBL (A) *FOXO1* expression negatively correlates with *MYC* and *JAK2* mRNA levels in PMBL samples. The data of 31 PMBL cases were mined from GEO database (GSE11318). We used following probe sets for correlation analysis: *FOXO1* - 202724_s_at; *JAK2* - 205842_s_at; *MYC* - 202431_s_at. The criteria for the chosen probe sets are described in “Material and Methods”. The data are shown as log2 of fluorescence intensity. Expression of *MYC* (B) and *JAK2* (C) in PMBL cell lines was measured by Q-RT-PCR. The data are shown as ratio to the reference gene *RPL13A*. (D) Expression of JAK2 and MYC protein in PMBL cell lines was analyzed by immunoblot. JAK2 and MYC expression was normalized to MedB-1 cells. (E). PMBL cell lines were treated for 24 h with the JAK2 inhibitor TG101348. FOXO1 expression was measured by Q-RT-PCR and by immunoblot. Statistical analysis was done by a two-way t-test. FOXO1 expression in the control group was compared with groups treated with increasing concentrations of the inhibitor. For immunoblot ACTB was used as a loading control. To control repression of JAK2 activity we used anti-phospho-STAT6 antibody. (F) PMBL cell lines were incubated with the MYC inhibitor 10058-F4 for 24h. FOXO1 expression was measured by Q-RT-PCR. The data were analyzed by comparative Ct method.

We hypothesized that FOXO1 down-regulation in PMBL may result at least in part from transcriptional repression. To find factors that might be responsible for the repression of *FOXO1*, we mined available GEP data of 31 PMBL cases to identify genes whose expression negatively correlates with *FOXO1* expression (Table S2). Among these genes was *JAK2*. Given that JAK2 inhibition blocks FOXO1 nuclear export [30] and due to the critical role of JAK2 in survival and proliferation of PMBL and cHL cell lines [8] we used correlation analysis to prove the correlation between *FOXO1* and *JAK2* expression. We actually found a significant negative correlation between *JAK2* and *FOXO1* in PMBL samples (Figure 4A). Since JAK2 maintains proliferation of PMBL and cHL cell lines by induction of MYC [8], we also investigated correlation between these genes. In fact, we found a positive correlation between *MYC* and *JAK2* (Figure 4A). Given reciprocal interactions between FOXO1 and MYC [31], we assessed their expression and found a significant negative correlation (Figure 4A). Next, we asked whether the correlations between *FOXO1, JAK2*, and *MYC* expression, found in primary PMBL tumors also holds true in PMBL cell lines. Therefore, we measured the mRNA and protein expression levels of these three factors in MedB-1, Karpas1106, and U2940 cells by Q-RT-PCR and by immunoblot. The expression of *MYC* and *JAK2* positively correlated at the mRNA level in all cell lines confirming the results from the primary cases. Moreover, a positive correlation was observed at the protein level where the highest expression of both genes was detected in MedB-1 cells (Figure 4B,C,D). Like in PMBL primary cases, expression of FOXO1 inversely correlated with expression of MYC and JAK2 in PMBL cell lines (Figure 2A,B and Figure 4B,C,D). Thus, PMBL cell lines are similar to original tumors in terms of *FOXO1, JAK2*, and *MYC* expression. Since *JAK2* and *FOXO1* correlated negatively, we investigated whether inhibition of JAK2 activity is able to increase FOXO1 levels. The physiological effect of JAK2 inhibition was controlled by analyzing the phosphorylation status of STAT6, a known JAK2 target in PMBL [32].

In all cell lines treatment with JAK2 inhibitor mildly increased *FOXO1* mRNA expression (Figure 4E). Interestingly, this weak transcriptional upregulation resulted in upregulation of FOXO1 protein level in MedB-1 and U2940, but not in Karpas1106 cell line. The efficiency of JAK2 inhibition by TG101348 was proven by a concentration-dependent decrease of STAT6 phosphorylation in all cell lines (Figure 4E). Given reciprocal interactions between MYC and FOXO1 we asked whether MYC downregulation might also increase FOXO1 expression. We found that the small molecular weight inhibitor 10058-F4 induced a significant and dose-dependent upregulation of *FOXO1* mRNA specifically in MedB-1 cells (Figure 4F). Taken together our results suggest that the JAK2/MYC axis contributes to FOXO1 repression in PMBL.

### FOXO1 is a tumor suppressor in PMBL

**Figure 5:**
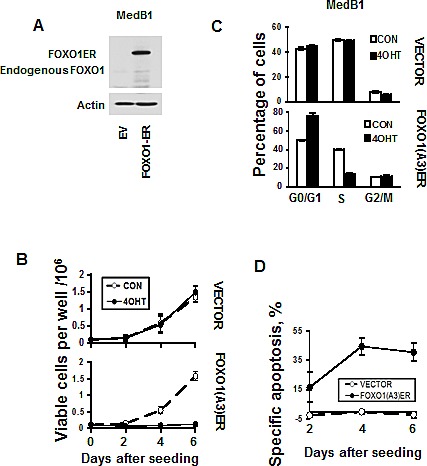
FOXO1 inhibits growth and induces apoptosis in MedB-1 cells (A) Expression of FOXO1-ER fusion protein and endogenous FOXO1 in MedB-1 cells transduced with constitutively active FOXO1 variant or with empty vector (EV) was analyzed by immunoblot. (B) MedB-1 cells transduced with FOXO1ER or with empty vector were seeded in six-well plates at a density of 1×10^5^ cells in 3 ml of complete culture medium. The next day EV and FOXO1ER cells were treated with 200 nM 4-OHT or with vehicle. Live cells were count by hemacytometer according to morphological criteria. The data of one of three independent experiments that yielded similar results are shown as mean±SD. All measurements were done in triplicate. (C) FOXO1 induces G_1_ arrest. The cell cycle distribution was measured 48 h after treatment with 4-OHT using PI staining. The data are shown as mean±SD of three measurements. Results of one of three independent experiments that yielded similar results are shown (D) Cell were seeded and treated with 4-OHT as it was described for (A). Cell death was measured by annexin V/7-AAD staining simultaneously with cell counting. The specific apoptosis (SA) was calculated as: SA(%) = 100 × (A_E_ − A_C_)/(100 − A_C_), where A_E_ equals the percentage of apoptotic cells in the experiment group and A_C_ equals percent of apoptotic cells in the control group. The measurements were done in triplicate by using flow cytometry. The results of one of three independent experiments that yielded similar results are shown as mean of SA±SD.

To investigate the functional role of FOXO1 repression in PMBL we overexpressed a constitutively active inducible variant of *FOXO1* (*FOXO1ER*) in the FOXO1 negative cell line MedB-1 (Figure 5A). Activation of FOXO1 by 4-OHT led to a decrease of cell viability in comparison to untreated control. 4-OHT did not influence proliferation of MedB-1 cells expressing empty vector (Figure 5B). The decrease of viable cells can be explained by a block of proliferation due to accumulation of cells in G_1_-phase (Figure 5C). FOXO1-induced cell death was associated with activation of caspase 3 (Figure 5D and Figure S3). Therefore, FOXO1 repression in PMBL contributes to the maintenance of the oncogenic program in this lymphoma type.

### FOXO1 activation represses genes responsible for proliferation and survival in PMBL

**Figure 6:**
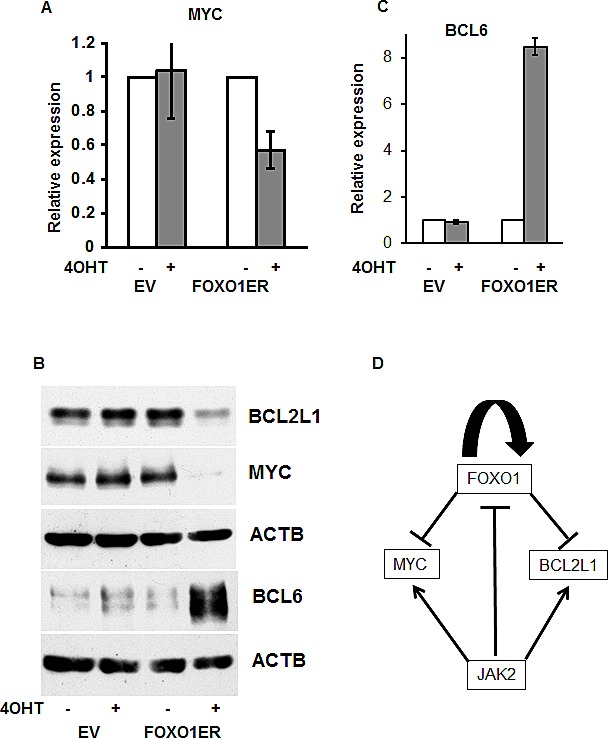
The antitumor effect of FOXO1 is associated with inhibition of MYC and BCL2L1 (A,B,C) MedB-1 cells expressing FOXO1ER or empty vector (EV) were treated with 200 nM 4-OHT. After 24 h incubation with 4-OHT or vehicle, cells were harvested and used for mRNA or protein isolation. (A) *MYC* repression was analyzed by Q-RT-PCR. (B) Repression of MYC and BCL2L1, and upregulation of BCL6 was analyzed by immunoblot. ACTB was used as loading control. (C) *BCL6* mRNA induction by FOXO1 was measured by Q-RT-PCR. (D) Model depicting the role of FOXO1 repression in maintaining of PMBL.

Given that proliferation of PMBL depends on expression of the protooncogene *MYC* [8], and that constitutive JAK-STAT activation is responsible for expression of the antiapoptotic gene *BCL2L1* [33] in PMBL, we analyzed the effect of FOXO1 on expression of MYC and BCL2L1 in MedB-1 cells. As a control for physiological effects of FOXO1 we measured the expression of the known direct FOXO1 target gene *BCL6* [34]. FOXO1 activation almost completely abolished MYC protein expression, although *MYC* mRNA levels were only moderately affected (Figure 6A,B). Expression of BCL2L1 protein was also strongly downregulated (Figure 6B), while BCL6 was upregulated. (Figure 6C,B). Therefore, repression of MYC and BCL2L1 might contribute to the tumor suppressor effects of FOXO1 in PMBL.

## DISCUSSION

We found that FOXO1 is downregulated at both the mRNA and protein levels in PMBL. In 95% of PMBL cases analyzed by IHC FOXO1 expression was low or absent, and in all samples analyzed by Q-RT-PCR *FOXO1* mRNA was expressed at substantially lower levels than in normal B cells. Several functional mechanisms including repression by JAK2 and MYC, as well as deregulation of a positive feedback mechanism regulating *FOXO1* promoter activity, contribute to FOXO1 repression in PMBL. Activation of FOXO1 in MedB-1 cells led to growth arrest and apoptosis which was associated with repression of the protooncogene MYC and the antiapoptotic protein BCL2L1 (Figure 6D).

PMBL is similar to cHL in molecular aspects [3, 4]. Therefore, downregulation of FOXO1 in PMBL provides additional evidence of the close similarities of cHL and PMBL oncogenic programs. At the same time our finding raises the question of heterogeneity of PMBLs. Although virtually all PMBL expressed FOXO1 mRNA and protein at lower levels than normal B cells, there were substantial differences among primary cases and cell lines. In PMBL complete repression of FOXO1 was observed only in 15 % of cases, whereas only 3% of the cases were FOXO1-positive in cHL [11]. The observed heterogeneity in FOXO1 expression is in line with reported morphologic and molecular differences among PMBL cases [35, 36]. Of note, this heterogeneity cannot be explained by genetic aberrations. We did not find *FOXO1* mutations in PMBL cell lines. We have proved that chromosomal aberrations are not responsible for FOXO1 repression in PMBL, therefore our data are in line with the recent next-generation sequencing study which also did not reveal *FOXO1* genetic aberrations in 10 primary PMBL cases [37]. Epigenetic silencing is certainly involved in FOXO1 repression in some cases of PMBLs but it is likely to play an indirect role because we were not able to detect methylation of the promoter CpG-island in the FOXO1-negative cell line. Interestingly, although FOXO1 is frequently repressed in different tumor types, promoter hypermethylation has never been observed by other studies [38, 39].

We identified JAK2 as a factor contributing to FOXO1 repression in PMBL. The ability of JAK2 to repress FOXO was shown in different cellular models. In neuronal cells JAK2 activation led to nuclear export of FOXO1 [30]. Mechanistically, the repression of FOXO1 can be explained by activation of PI3K-AKT and ERK pathways by JAK2 [40]. This, in turn, results in phosphorylation, nuclear export, and degradation of FOXO transcription factors [17, 41]. It is conceivable that the upregulation of FOXO1 protein by the JAK2 inhibitor is due to repression of FOXO1 inactivating pathways. The increase in *FOXO1* transcription by repression of JAK2 and the negative correlation between *JAK2* and *FOXO1* mRNA expression in PMBL samples might be explained by positive autoregulatory feedback loop controlling *FOXO1* transcription [29].

We further observed that a small molecular weight MYC inhibitor reactivates *FOXO1* transcription in the FOXO1-negative cell line MedB-1. Considering that MYC was shown to compete with FOXO3 for PUMA, GADD45A, and p27/CDKN1B promoters [15, 42], it is conceivable that MYC can also interfere with FOXO1 binding on the *FOXO1* promoter thus disrupting a positive autoregulatory mechanism.

FOXO transcription factors simultaneously activate numerous proapoptotic and antiproliferative pathways. Therefore it is hard to determine single factors responsible for FOXO-induced cell death and growth arrest [15]. In the present study we analyzed only FOXO targets which are known to play a critical role in the maintenance of PMBL. We found that FOXO1 represses the protooncogene MYC and inhibitor of apoptosis BCL2L1 in MedB-1 cells. FOXO proteins were shown to repress MYC at the mRNA and protein levels by induction of *miR-145, miR-34b*, and *miR-34c* [43, 44]. In addition, FOXO3 inhibits MYC protein stability by decreasing phosphorylation [45]. Given that FOXO1 activation in MedB-1 cells led to strong inhibition of MYC protein but relatively mild reduction of *MYC* mRNA level, the inhibition of translation and protein stability might be critical.

BCL2L1 is an indirect repression target of FOXO proteins. FOXO4/AFX protein induced death of HeLa cells through upregulation of BCL6, the repressor of BCL2L1 [46]. Considering that FOXO1 strongly activated BCL6 protein expression, this mechanism might contribute to BCL2L1 repression in MedB-1 cells. Interestingly, expression of BCL2L1 in GC-DLBCL (bcl6+/CD10+/-/MUM1-/CD138-) was significantly lower than in non-GC-DLBCL (bcl6-/CD10-/MUM1+/CD138-)[47].

We have shown that FOXO1 repression contributes to the maintenance of PMBL and that constitutive expression of JAK2 and MYC is involved in FOXO1 downregulation. We identified the protooncogene *MYC* and the antiapoptotic gene *BCL2L1* as FOXO1 repression targets in PMBL.

## MATERIAL AND METHODS

### Cell lines, chemicals, and treatments

PMBL cell lines MedB-1, Karpas1106, and U2940, were cultured in RPMI 1640 medium supplemented with 10% FCS, glutamine, and antibiotics as described [23]. MedB-1 cells stably expressing a constitutively active form of human FOXO1 in-frame with the modified tamoxifen-specific version of the murine estrogen receptor-α ligand-binding domain (FOXO1-ER) were established by infection with pCFG5-FOXO1(A3)ER retroviral vector followed by selection with 100 μg of zeocin as we described earlier [11]. Control cells were transduced with empty pCFG5-IEGZ vector. FOXO1ER construct was induced by 4-hydroxytamoxifen (4-OHT) (Merck Millipore, Schwalbach, Germany) at a final concentration of 200 nM. The small-molecular weight MYC inhibitor 10058-F4 was obtained from Sigma-Aldrich (Steinheim, Germany). The JAK2 inhibitor TG 101348 was obtained from Axon Medhem (Groningen, The Netherlands); 5-aza-dC and TSA were purchased from Calbiochem (Darmstadt, Germany).

### DNA methylation and mutational analysis

For analysis of the methylation status of the *FOXO1* promoter CpG island we used pyrosequencing [11]. The mutational status of *FOXO1* coding exons was analyzed by direct sequencing as we described earlier [11].

### Cell death and proliferation analysis

Apoptosis was assessed by flow cytometry using annexin V-PE and 7-amino-actinomycin D co-staining [11]. For cell-cycle analysis we used PI staining [11].

### Quantitative RT-PCR

Total RNA was isolated and first strand cDNA was synthesized as described [11]. Samples were amplified with help of IQ^™^SYBR Green Supermix (BIO-RAD, Munich, Germany) using LightCycler 480 real-time PCR instrument (Roche Diagnostics, Mannheim, Germany). We used following primer sets: FOXO1: 5′-tggacatgctcagcagacatc-3 and 5′-ttgggtcaggcggttca-3′; FOXO1 3′-UTR: 5′- cccattgtgtgttgaaatcc-3 and 5′-ttgctttccagacagaccag-3′; MYC: 5′- tcggattctctgctctcctc-3′ and 5′- tgttcctcctcagagtcgct-3′; RPL13A: 5′-cggaccgtgcgaggtat-3′, and 5′-caccatccgctttttcttgtc-3′; BCL6: 5′-agagcccataaaacggtcct -3 and 5′-tggtccacaacagtctcca-3′; JAK2: 5′-tttggcaacagacaaatgga-3′and 5′-gcaggaagctgatgcctatc-3′. Annealing temperature was 60 ºC for all primers. Primer sequences were identified using Genscript online software (www.genscript.com, 12.08.2013). All oligonucleotides were synthesized by biomers.net (Ulm, Germany).

### Immunoblot

Immunoblot was done as described earlier [24]. The following primary antibodies were used: anti-FOXO1 rabbit # 2880 (Cell Signaling Technology, Danvers, MA); anti-MYC rabbit sc-788 (Santa Cruz Biotechnology, Heidelberg, Germany); anti-JAK2 sc-278 (Santa Cruz); anti-beta-Tubulin ab6046 (Abcam, Cambridge, UK); anti-actin rabbit A5060 (Sigma-Aldrich). As second antibody we used goat anti–rabbit IgG-HRP (sc-2004; Santa Cruz). Signals were visualized using the SuperSignal West Dura extended-duration substrate (Thermo Scientific).

### Human material, immunohistochemistry

Twenty cases of PMBL were included in this study. Lymphoma diagnosis was in accordance with the current World Health Organization classification [10]. As control, we used samples of non-neoplastic tonsils. All PMBL samples were drawn from our archive of formalin-fixed, paraffin-embedded tissues and pseudonymized to comply with the German law for ethical usage of archival tissue for clinical research (Deutsches Ärzteblatt 2003; 100 A1632). Approval for these studies was obtained from the University of Ulm ethics board. The CD19^+^ tonsillar cells and B-cell subtypes were isolated as described earlier [25]. For immunostaining, deparaffinized tissue sections were heat-denatured in a pressure cooker and incubated with rabbit monoclonal antibody against FOXO1 used for immunoblot (1:25 dilution). Bound antibody was labeled using EnVision System (Dako, Jena, Germany). Peroxidase activity was visualized by the substrate 3-amino-9-ethylcarbazole (0.1 mg/mL in 0.17 M sodium acetate, pH 5.2 plus 0.01% H_2_O_2_). The images were acquired as described earlier [11].

### Gene expression and DNA copy number analysis

Gene expression profiling and CGH data of 31 PMBL cases were mined from GEO database (http://www.ncbi.nlm.nih.gov/geo/; GSE 11318; 20.08.2013). Gene expression data were analyzed with help of Genesifter software (Perkin Elmer, Seattle, WA). Copy number aberrations were detected using the R package on the original publication by Olshen and Venkatraman [26].

### Correlation analysis of FOXO1, MYC, and JAK2 expression

The data were mined from GEO database (GSE11318; http://www.ncbi.nlm.nih.gov/geo/, 10.08.2013). To analyze *FOXO1* expression we used the probe set 202724_s_at. In our pilot experiments we found that *FOXO1* expression levels in B-cell lymphomas and B cell subtypes (GEO data set GDS3516, 18.02.2011) assessed with help of 202724_s_at and 202723_s_at probe sets perfectly correlated with the results of our Q-RT-PCR, immunoblot, and immunohistochemistry in the relevant tissues [11]. We have chosen 202724_s_at probe set because it had highest Jetset score (overall score 0.443). The scoring is based on assessment of specificity, coverage of all transcripts, and probability of transcription of target [27]. The *MYC* probe set 202431_s_at we used was the only true *MYC* probe set on Affymetrix Human Genome U133 Plus 2.0 Array (overall score 0.328). *JAK2* is represented by two probe sets 205842_at and 205841_at. Both *JAK2* probe sets revealed a statistically significant negative correlation between *FOXO1* and *JAK2* expression. When we assessed correlation between *MYC* and *JAK2* expression both probe sets revealed a positive correlation, but only 205842_at yielded statistically significant results and was chosen for analysis. Statistical significance of the correlation was measured by Statistics Calculators software (http://www.danielsoper.com/statcalc3/calc.aspx?id=44; 13.08.2013).

## SUPPLEMENTARY INFORMATION FIGURES AND TABLES


