# Supplementary material for: FOXO1 downregulation contributes to the oncogenic program of primary mediastinal B-cell lymphoma
Source: Oncotarget. 2014 Jun 15;5(14):5392–402. doi: 10.18632/oncotarget.2107 (PMC4170625; doi:10.18632/oncotarget.2107)
Supplement: Supplementary file 1 [file oncotarget-05-5392-s001.pdf]

FOXO1 downregulation contributes to the oncogenic program of primary mediastinal B-cell lymphoma

Supplementary Information

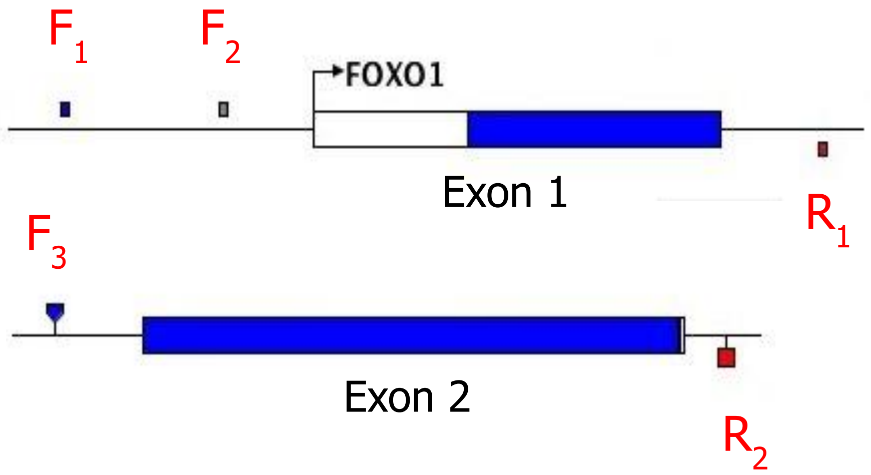

Figure S1: Structure of *FOXO1* gene and location of sequencing primers. The sequence of *FOXO1* gene was mined from NCBI database (http://www.ncbi.nlm.nih.gov/gene/2308; 12.08.2013) and visualized by using GenePalette software (http://www.genepalette.org). The primer sequences were found with help of "Sequencing primer design" on-line utility (Genescript, http://www.genscript.com, 13.08.2013). Genomic DNA was isolated from normal B cells and from PMBL cell lines using DNeasy Tissue Kit (Qiagen, Hilden, Germany) and amplified with help of Phusion High-Fidelity DNA polymerase (New England BioLabs). Both forward and reverse primers were used for amplification. Exon 1 was amplified with primers F1: 5'-ATCGCAGCGAAAGAAAACAT-3' and R1 5'-CTTCTTGGCGCACTTTCTTT-3' and exon 2 was amplified with primers: F3: 5'-ACCGCACCCAGCCACAGATA -3' and R2: 5'-GATCCCTCTCTGCAAGGGACAG-3'. The PCR product was purified by agarose gel electrophoresis followed by extraction of the amplified product with help of Gel extraction kit (Qiagen). For sequencing we used the same primers as for amplification with exception of sequencing primer F2: 5´-TGTGTGAAAAACACCC-3 because of the long poly A tract located downstream of F1 primer. Long homopolymer stretches of DNA sequence cannot be reliably sequenced because of polymerase shutter. The purified amplification products were sequenced by GATC biotech (Constance, Germany). The sequences were analyzed with help of Lasergene software (DNASTAR).

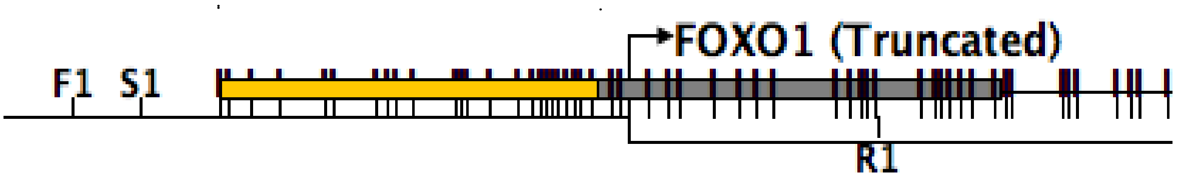

Figure S2: Methylation status of *FOXO1* promoter CpG island in MedB-1 cell line. Structure of FOXO1 promoter. F1 and R1 - forward and reverse amplification primers, respectively; S1 – sequencing primer. CpG dinucleotides are shown as vertical dashes; CpG island is shown as a horizontal bar. The analyzed 26 CpGs are shown in yellow. Methylation status of FOXO1 promoter in tonsillar tissue obtained from different patients, CD19+ tonsillar cells, CD19+/CD10+ germinal center B cells, and MedB-1 cell line was analyzed by pyrosequencing. gDNA was isolated and bisulfite converted with help of EZ DNA Methylation-DirectTM Kit (Fa. Zymo Research). The converted gDNA was amplified by PyroMark PCR Kit (Qiagen). The sequence of primers: F1: 5´-GGA GGT GAG TGT GTG AAA GA-3´; R1: 5´-biotin-AAC CCC TTA CTC TCC CCA AC-3´; S1: 5´-AAT ATT TTA TTA TTT TTT ATT-3´. The sequences of the primers were found with help of PyroMarkAssay Design 2.0 Software (Qiagen). The methylation status of *FOXO1* promoter CpG island was analyzed with help of the PSQ96MA pyrosequencer (Qiagen).

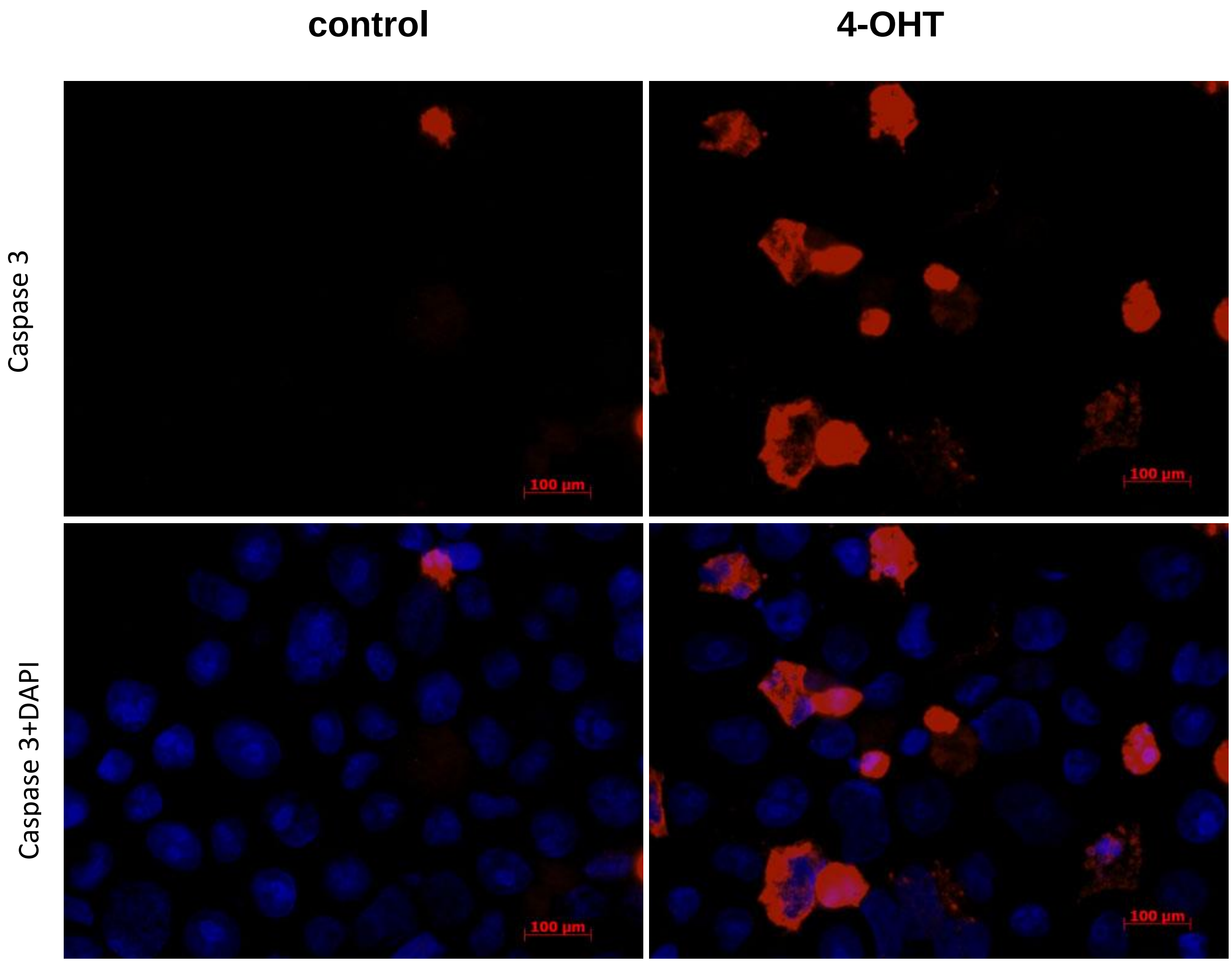

Figure S3: FOXO1 activates caspase 3 in the PMBL cell line MedB-1. Cells were treated with 200 nM 4-OHT or with vehicle and were harvested 48h after treatment. Cells were attached to object slides by centrifugation in a cytospin centrifuge, fixed with a mixture of methanol and acetone (1:1) at -20 °C and blocked in filtered 0.1% PBS-skimmed milk for 1 h at room temperature. Then they were incubated with rabbit anti-human cleaved CASP3 antibody (Dako, Hamburg, Germany) followed by donkey anti-rabbit AlexaFluor-594 antibody (Invitrogen, Life Technologies, Darmstadt, Germany) and DAPI (Merck, Darmstadt, Germany). The slides were mounted in Mowiol and images were captured by AxioVert 200 Mimicroscope (Zeiss) equipped with AxioCamMRm camera with help of AxioVision Rel. 4.8 software (Zeiss). Objective: Zeiss Plan APO 63x. CASP3 and DAPI expression are shown as RED and BLUE, respectively.

Table S1: Aberrant segments affecting *FOXO1* in 5 PMBL cases.

ID	Chromosome	Aberration startpoint (bp)	Aberration endpoint (bp)	Number of markers	Segment mean ratio (log2)
GSM286982	13	40701168	43215010	368	0.2834
GSM286986	13	34884419	48526505	1952	-0.1443
GSM286991	13	31915231	68893868	5025	0.2437
GSM286999	13	17918051	114125831	13359	0.3572
GSM287006	13	17918051	114125831	13359	0.2847

Gene expression profiling and CGH data of PMBL cases were mined from GEO database (http://www.ncbi.nlm.nih.gov/geo/; GSE 11318; 20.08.2013). Dataset „Molecular subtypes of DLBCL have different chromosomal aberrations".Copy number aberrations were detected using the R package.

Table S2:Top 40 genes negatively correlating with *FOXO1* expression in PMBL

Other ID	Gene Title	Gene ID
203418_at	Cyclin A2	CCNA2
219207_at	Enhancer of mRNA decapping 3 homolog (S. cerevisiae)	EDC3
220800_s_at	Tropomodulin 3 (ubiquitous)	TMOD3
242977_at	Hypothetical LOC400796	LOC400796
240700_at	Transcribed locus, strongly similar to NP_659471.1 torsin A interacting protein 2 [Homo sapiens]	-
210570_x_at	Mitogen-activated protein kinase 9	MAPK9
207450_s_at	POU class 6 homeobox 2	POU6F2
228222_at	Transcribed locus	-
216901_s_at	IKAROS family zinc finger 1 (Ikaros)	IKZF1
1558661_at	Homo sapiens, clone IMAGE:5539086, mRNA	-
203466_at	MpV17 mitochondrial inner membrane protein	MPV17
200693_at	Tyrosine 3-monooxygenase/tryptophan 5-monooxygenase activation protein, theta polypeptide	YWHAQ
225653_at	Hypothetical LOC644617	LOC644617
211584_s_at	Nuclear protein, ataxia-telangiectasia locus	NPAT
231905_at	chromosome 20 open reading frame 96	-
223177_at	5'-nucleotidase domain containing 1	NT5DC1
1555785_a_at	5'-3' exoribonuclease 1	XRN1
218512_at	WD repeat domain 12	WDR12
209249_s_at	Growth hormone inducible transmembrane protein	GHITM
217307_at	aconitase 2, mitochondrial	-

1557360_at	Transcribed locus	-
201151_s_at	Muscleblind-like (Drosophila)	MBNL1
240950_s_at	Coiled-coil domain containing 155	CCDC155
207381_at	Arachidonate 12-lipoxygenase, 12R type	ALOX12B
209748_at	Spastin	SPAST
1554691_a_at	ADP-ribosylation factor GTPase activating protein 3	ARFGAP3
1558922_at	Full length insert cDNA clone YF43G08	-
1568751_at	regulator of G-protein signaling 13	RGS13
200793_s_at	Aconitase 2, mitochondrial	ACO2
239526_x_at	Protein tyrosine phosphatase, non-receptor type 1	PTPN1
202543_s_at	Glia maturation factor, beta	GMFB
201263_at	threonyl-tRNA synthetase	-
223078_s_at	Tropomodulin 3 (ubiquitous)	TMOD3
205842_s_at	Janus kinase 2 (a protein tyrosine kinase)	JAK2
200970_s_at	Stress-associated endoplasmic reticulum protein 1	SERP1
228559_at	Centromere protein N	CENPN
234117_at	Asthma-associated alternatively spliced gene 1	AAA1
1560748_at	Homo sapiens, clone IMAGE:5721950, mRNA	-
220881_at	Homo sapiens hypothetical protein PRO1787 (PRO1787), mRNA.	-
1558119_at	Tetraspanin 17	TSPAN17

The data were reanalyzed with help of GeneSifter software to find genes whose expression negatively correlates with *FOXO1*. Normalization: RMA. Analysis type: “Genesifter “Search by gene pattern”. Method: centered. Correlation coefficient: -0.45. FOXO1 probe set 202724_s_at was used for this analysis. GEO data set GSE 12453.
